# Supplementary material for: Curated and harmonised transcriptomics datasets of interstitial lung diseases
Source: Data Brief. 2025 Oct 14;63:112139. doi: 10.1016/j.dib.2025.112139 (PMC12581653; doi:10.1016/j.dib.2025.112139)
Supplement: Supplementary file 1 [file mmc1.zip › Supplementary_material/RNA-seq/fastQC reports/GSE185492/TRIMMED_SRR16230231_32_fastqc.html]

TRIMMED\_SRR16230231\_32.fastq.gz FastQC Report 

FastQC Report

Wed 18 Jan 2023  
TRIMMED\_SRR16230231\_32.fastq.gz

## Summary

- Basic Statistics
- Per base sequence quality
- Per sequence quality scores
- Per base sequence content
- Per sequence GC content
- Per base N content
- Sequence Length Distribution
- Sequence Duplication Levels
- Overrepresented sequences
- Adapter Content

## Basic Statistics

| Measure | Value |
| --- | --- |
| Filename | TRIMMED\_SRR16230231\_32.fastq.gz |
| File type | Conventional base calls |
| Encoding | Sanger / Illumina 1.9 |
| Total Sequences | 34592754 |
| Sequences flagged as poor quality | 0 |
| Sequence length | 60-100 |
| %GC | 48 |

## Per base sequence quality

## Per sequence quality scores

## Per base sequence content

## Per sequence GC content

## Per base N content

## Sequence Length Distribution

## Sequence Duplication Levels

## Overrepresented sequences

No overrepresented sequences

## Adapter Content

Produced by FastQC (version 0.11.7)
